# Associations among health literacy, anxiety symptoms, and health-related quality of life in Korean adults: A cross-sectional study with age-stratified analyses

**DOI:** 10.1371/journal.pone.0342239

**Published:** 2026-02-09

**Authors:** Gyuri Seol, Young Hwangbo, Yongbae Kim, Youngs Chang, Mee-Ri Lee

**Affiliations:** 1 Department of Medical Science, Soonchunhyang University, Cheonan-si, Republic of Korea; 2 Department of Preventive Medicine, Soonchunhyang University College of Medicine, Cheonan-si, Republic of Korea; Xiamen University - Malaysia Campus: Xiamen University - Malaysia, MALAYSIA

## Abstract

**Background:**

Health literacy (HL) is a key determinant of physical and mental health outcomes; however, the relationships among HL, anxiety symptoms, and health-related quality of life (HRQoL) remain unclear, and whether the effects of HL vary by age is unknown. We aimed to examine the associations among HL, anxiety symptoms, and HRQoL in Korean adults and assessed age-related differences in these associations.

**Methods:**

In this cross-sectional study, we analyzed data from the 2023 Korea National Health and Nutrition Examination Survey, including 5,017 adults aged ≥ 19 years. HL was assessed using a validated 10-item instrument (score range: 10–40) and categorized as low, middle, or high. Anxiety symptoms and HRQoL were measured using the 7-item Generalized Anxiety Disorder Scale and the 8-item Health-related Quality of Life Instrument, respectively. Multivariable logistic regression models adjusted for potential confounders were used to estimate associations between HL and anxiety symptoms and between HL and good HRQoL. Age-stratified analyses were conducted for participants aged 19–39, 40–64, and ≥ 65 years.

**Results:**

The low (odds ratio [OR]: 1.93; 95% confidence interval [CI]: 1.52–2.46; p < 0.001) and middle HL (OR: 1.30; 95% CI: 1.04–1.62; p = 0.024) groups had higher odds of anxiety symptoms than the high HL group. Lower HL was associated with a reduced likelihood of good HRQoL (OR: 0.49; 95% CI: 0.36–0.66; p < 0.001), whereas the middle HL group showed a non-significant trend toward poorer HRQoL (OR: 0.77; 95% CI: 0.56–1.06). HL was associated with anxiety symptoms in young and middle-aged adults, and with HRQoL in young and older adults.

**Conclusion:**

Low HL was significantly associated with increased anxiety symptoms and poor HRQoL, with a significant impact among young adults. These findings highlight the need for age-specific public health strategies to improve HL.

## Introduction

Health literacy (HL) refers to an individual’s ability to access, understand, appraise, and apply health-related information and services to make appropriate health decisions and take informed actions. The World Health Organization (WHO) characterizes HL as a stronger predictor of health status than socioeconomic factors, such as income and educational attainment [1]. Higher HL is associated with effective personal health management, improved health indicators, and broader social benefits [2]. In contrast, limited HL is associated with poorer health outcomes, including worse overall health status [3], reduced use of preventive services [4], more frequent hospital admissions [5], and increased mortality [6]. Despite its importance low HL remains common across populations. In Korea, approximately 55.4% of adults have been classified as having limited HL [7], indicating a significant public health concern, even within a highly educated society [8].

In the United States, nearly half of adults have limited HL [9]. In Europe, at least 12% of respondents demonstrate insufficient HL, and almost half (47%) are classified as having limited HL, defined as insufficient or problematic HL [10]. Limited HL may hinder access to health information and contribute to delayed care and increased anxiety.

Low HL is associated with a higher prevalence of anxiety symptoms [11,12]. However, most previous studies have focused on mental HL, and only a few have directly examined the association between HL and anxiety symptoms [13,14]. Therefore, evidence clarifying the relationship between these two variables remains limited [15,16].

In addition to mental health outcomes, HL may influence perceived health-related quality of life (HRQoL) [17,18]. HRQoL reflects an individual’s perception of quality of life within cultural and social contexts [19] and is widely used to evaluate the impact of medical interventions and population-based health surveys [20]. Higher HL has been associated with better HRQoL [21,22]; however, findings remain inconsistent. For example, a longitudinal study of Canadian patients with type 2 diabetes reported that lower HL was associated with poorer HRQoL [23]. In contrast, a study of Chinese patients with chronic heart failure found no significant association between these variables [24]. These inconsistencies may reflect differences in study populations. Previous research has primarily focused on patients with specific conditions or on particular age groups, such as older adults, adolescents, and young adults [17,18]. In addition, some studies were conducted in specific contexts, such as during the Coronavirus Disease 2019 (COVID-19) pandemic [16,25], which may limit the generalizability of their findings. To date, studies examining the simultaneous effects of HL on anxiety symptoms and HRQoL remain scarce, particularly in general adult populations [26–28].

Furthermore, the relationship between HL and age remains unclear. Some studies have reported lower HL among older adults [29,30], whereas others have observed no clear age-related trend or even lower HL among younger individuals [31,32]. These inconsistencies highlight the need to clarify how HL influences mental health and the quality of life across different age groups.

Therefore, in this current study, we utilized data from the 2023 Korea National Health and Nutrition Examination Survey (KNHANES) to examine differences in anxiety symptoms and HRQoL according to HL levels among Korean adults. Furthermore, we assessed whether the associations among HL, anxiety symptoms, and HRQoL varied by age. We aimed to provide empirical evidence on the relationship between HL, mental health, and quality of life and to generate foundational data to inform the development of age-specific interventions to improve HL and overall well-being.

## Conceptual framework

**Fig 1:**
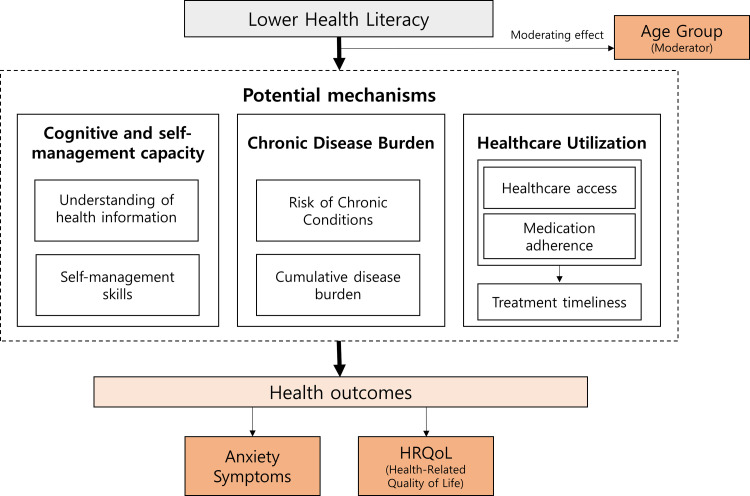
Conceptual framework illustrating hypothesized pathways linking health literacy to anxiety symptoms and health-related quality of life (HRQoL).

This study was guided by a conceptual framework outlining the hypothesized relationships among HL, anxiety symptoms, and HRQoL (Fig 1). We hypothesized that lower HL is associated with greater anxiety symptoms and poorer HRQoL through several potential mechanisms, including difficulty understanding and applying health information, reduced self-management capacity, a higher burden of chronic disease, and differences in healthcare utilization. Age was conceptualized as an effect modifier, such that the strength and relevance of these pathways may vary across young, middle-aged, and older adults. This framework provides the rationale for the age-stratified analyses and supports interpretation of the observed associations in this cross-sectional study.

This framework depicts potential mechanisms through which health literacy may influence anxiety symptoms and HRQoL, including cognitive and self-management capacity, chronic disease burden, and healthcare utilization, based on prior literature. Age group is conceptualized as a moderating factor that may influence these pathways. Arrows represent hypothesized relationships and do not imply causal or mediation effects.

## Materials and methods

### Study design

This study is a cross-sectional observational analysis based on data from the 2023 KNHANES. We conducted a secondary analysis of a nationally representative sample of Korean adults. HL was treated as the exposure variable, and anxiety symptoms and HRQoL were treated as outcome variables. Given the cross-sectional design, temporal relationships between HL and the outcomes could not be established. Because a fixed sample from the 2023 KNHANES was used in this study, an a priori sample size calculation was not feasible.

### Data collection and study population

This study was based on data from the second year of the ninth KNHANES cycle (2023), a nationally representative survey conducted annually by the Korea Disease Control and Prevention Agency (KDCA) under the National Health Promotion Act.

KNHANES employs a stratified, multistage probability sampling design to ensure representativeness of the Korean population aged 1 year and older. The survey comprises three components: health interviews, health examinations, and nutrition surveys. All procedures were approved by the relevant Institutional Review Board, and the public-use dataset was anonymized to protect participant confidentiality. We accessed the 2023 KNHANES data on February 7, 2025, through the KDCA.

**Fig 2:**
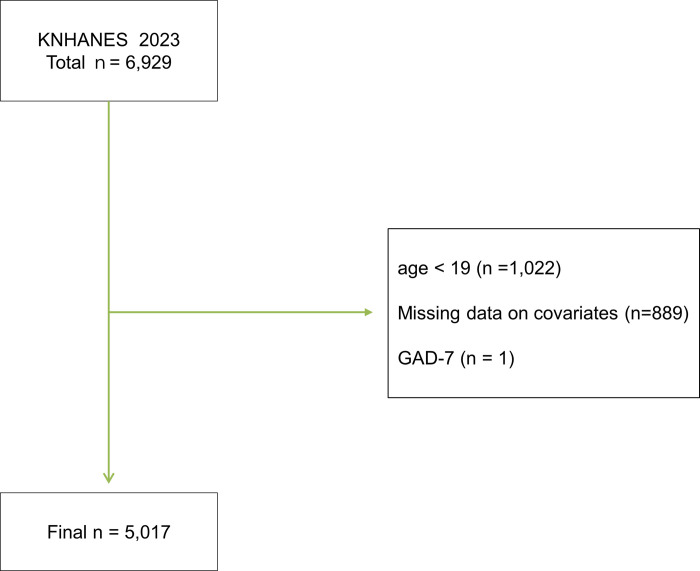
Flow diagram of participant selection for the study.

The initial sample included 6,929 participants. After excluding individuals younger than 19 years (n = 1,022) and those with missing data on covariates (n = 889) or the Generalized Anxiety Disorder-7 (GAD-7) scale (n = 1), the final analytical sample comprised 5,017 adults (Fig 2).

Flowchart showing the selection of study participants from the 2023 Korea National Health and Nutrition Examination Survey (KNHANES). Of the 6,929 participants initially assessed, those aged younger than 19 years (n = 1,022), those with missing covariate data (n = 889), and one participant with missing data on the Generalized Anxiety Disorder-7 (GAD-7) scale were excluded. The final analytical sample comprised 5,017 adults.

### Ethics statement

The 2023 KNHANES was conducted in accordance with the Declaration of Helsinki, and all participants provided informed consent.

We analyzed only anonymized, publicly available data.

The Institutional Review Board of the Soonchunhyang University Hospital approved the study protocol (IRB No. 2025-05-042-001).

## Exposure variable

### HL

The primary independent variable was HL, which was assessed using a validated 10-item instrument newly introduced in the 2023 KNHANES [33]. This instrument was developed specifically for the Korean healthcare context. Each item was rated on a 4-point Likert scale ranging from “strongly disagree” (1) to “strongly agree” (4). The instrument comprises four domains: disease prevention (three items), healthcare (four items), health promotion (one item), and resource utilization (two items). Total scores range from 10 to 40, with higher scores indicating higher HL. The instrument demonstrated high internal consistency (Cronbach’s α = 0.87). HL scores were classified into three levels based on cutoff points proposed in the original validation study of the KNHANES health literacy index [33]: low (≤ 28), middle (29–31), and high (≥ 32).

## Outcome variables

### GAD-7

Generalized anxiety symptoms during the past 2 weeks were assessed using the validated Korean version of the GAD-7 scale, a screening instrument used to identify individuals at risk for generalized anxiety disorder. Total scores range from 0 to 21, with higher scores indicating greater symptom severity. Consistent with previous KNHANES-based research, a cutoff score of ≥ 5 was used to define elevated anxiety symptoms [34,35].

### HRQoL

HRQoL was assessed using the Health-Related Quality of Life Instrument with eight Items (HINT-8), a preference-based instrument developed for the Korean population. The HINT-8 comprises eight items assessing multiple domains, including climbing stairs, pain, vitality, work performance, depressive mood, memory, sleep quality, and happiness. Each item is rated on a 4-point Likert scale. A utility index score ranging from 0 to 1 was calculated by applying preference weights to participant responses, with values closer to 1 indicating better quality of life [36]. In the KNHANES, HRQoL is evaluated using a biennial rotation of the EuroQol-5 Dimension instrument and the HINT-8. The HINT-8 was administered in the 2023 survey, and index scores were derived from participants’ HINT-8 responses [37]. Observed scores ranged from 0.303 to 0.927. In the absence of an established clinical cutoff, participants with scores in the lowest decile (< 0.688) were classified as having low HRQoL, consistent with previous research [38].

### Covariates

Several demographic and health-related variables were included as covariates. Age was categorized into three groups: 19–39 years, 40–64 years, and ≥ 65 years [39,40]. Other demographic variables included sex (male or female, with female as the reference category), marital status (married or unmarried), educational attainment (less than high school, high school graduate, or college graduate or higher), and household income (quartiles).

Female sex was selected as the reference category to maintain consistency with previous KNHANES-based studies and because women slightly outnumbered men in the study sample. Health behavior variables included smoking status (current smoker: defined as having smoked at least 100 cigarettes in one’s lifetime and currently smoking, or non-current smoker) and alcohol consumption (drinking ≥ once per month). Aerobic physical activity adherence was defined as engaging in ≥ 150 min of moderate-intensity physical activity per week, or ≥ 75 min of vigorous-intensity physical activity per week, or an equivalent combination of both [41].

Healthcare utilization and chronic disease burden were also considered. Outpatient visits within the past 2 weeks and inpatient hospitalizations during the past year were coded as “yes” or “no.” Chronic disease burden was quantified as the number of physician-diagnosed chronic conditions reported from a predefined list of eight common conditions: hypertension, diabetes mellitus, asthma, chronic rhinosinusitis, allergic rhinitis, obstructive sleep apnea, chronic kidney disease, and dyslipidemia [42]. Data on key mental health variables such as depression and perceived stress were not available in the 2023 survey cycle and therefore could not be included as covariates in the analysis.

### Statistical analyses

All statistical analyses were conducted in accordance with the KNHANES Analytic and Reporting Guidelines using a complex survey design. Sampling weights, stratification variables, and primary sampling units were applied in all analyses to account for the stratified, multistage probability sampling design of KNHANES. Descriptive statistics for participant characteristics and group comparisons were performed using the chi-square test.

For the primary analyses, HL was treated as a three-level categorical variable (low: ≤ 28; middle: 29–31; high: ≥ 32). Anxiety symptoms (GAD-7 ≥ 5) and HRQoL (HINT-8 < 0.688) were dichotomized using validated cutoffs.

Associations between HL and the outcomes (anxiety symptoms and HRQoL) were examined using multivariable logistic regression models to estimate odds ratios (ORs) with 95% confidence intervals (CIs). Models were adjusted for covariates described above, including age, sex, marital status, educational attainment, income quartile, smoking status, alcohol consumption, physical activity, chronic disease burden, and outpatient/inpatient health care utilization. Subgroup analyses were conducted to examine age-specific associations. All statistical tests were two-sided, and a p-value < 0.05 was considered statistically significant. Statistical analyses were performed using Stata version 14 (StataCorp, College Station, TX, USA). Multicollinearity among covariates was examined using variance inflation factors, and no significant problematic multicollinearity was detected.

## Results

### Study population characteristics

A total of 5,017 participants were included in the analysis (mean age: 53.3 ± 16.5 years; 43.1% male). HL levels were distributed as follows: low (n = 1,551), middle (n = 1,799), and high (n = 1,667).

Table 1 summarizes the general characteristics of the study population by HL level. HL differed significantly across age groups. Among participants in the 19–39 years age group, 56.1% were classified as having high HL. In contrast, among those aged ≥ 65 years, only 8.8% had high HL, whereas 33.4% had low HL.

**Table 1:** General characteristics of the study population according to the health literacy group.

	Low (N = 1,551)	Middle (N = 1,799)	High (N = 1,667)	Total (N = 5,017)	p-value
Age, years					**< 0.001**
19–39	322 (30.6)	494 (35.3)	781 (56.1)	1,597 (41.4)	
40–64	526 (36.0)	783 (44.1)	641 (35.1)	1,950 (38.5)	
≥ 65	703 (33.4)	522 (20.6)	245 (8.8)	1,470 (20.1)	
Sex					**< 0.001**
Male	716 (52.8)	790 (49.4)	657 (46.1)	2,163 (49.2)	
Female	835 (47.2)	1,009 (50.6)	1,010 (53.9)	2,854 (50.9)	
Education level					**< 0.001**
< High school	702 (35.4)	401 (17.2)	176 (7.0)	1,279 (18.7)	
High school	462 (33.7)	672 (37.5)	533 (32.9)	1,667 (34.7)	
≥ College	387 (31.0)	726 (45.4)	958 (60.1)	2,071 (46.5)	
Household income					**< 0.001**
Low	461 (24.2)	275 (11.9)	170 (8.6)	906 (14.3)	
Low-intermediate	399 (24.1)	457 (23.4)	338 (19.1)	1,194 (22.1)	
Intermediate	363 (26.5)	537 (32.3)	506 (31.9)	1,406 (30.5)	
Upper-intermediate	328 (25.2)	530 (32.4)	653 (40.4)	1,511 (33.2)	
Smoking					**< 0.001**
Yes	702 (49.6)	959 (56.6)	921 (58.8)	2,582 (55.4)	
No	849 (50.4)	840 (43.4)	746 (41.3)	2,435 (44.6)	
Alcohol consumption					**< 0.001**
Yes	498 (48.5)	1,312 (56.5)	772 (58.2)	2,582 (55.4)	
No	635 (51.5)	1,165 (43.5)	635 (41.8)	2,435 (44.6)	
Marital status					**< 0.001**
Married	1,310 (77.0)	1,518 (79.1)	1,242 (66.5)	4,070 (74.0)	
Unmarried	241 (23.0)	281 (20.9)	425 (33.5)	947 (26.1)	
Aerobic physical activity					**< 0.001**
Yes	573 (40.9)	821 (48.6)	898 (56.1)	2,292 (49.1)	
No	978 (59.1)	978 (51.4)	769 (43.9)	2,725 (50.9)	
Outpatient visits					0.225
Yes	432 (24.7)	499 (25.2)	397 (22.5)	1,328 (24.1)	
No	1,119 (75.3)	1,300 (74.8)	1,270 (77.5)	3,689 (75.9)	
Inpatient visits					0.449
Yes	169 (10.7)	190 (9.5)	156 (9.3)	515 (9.7)	
No	1,382 (89.3)	1,609 (90.5)	1,511 (90.7)	4,502 (90.2)	
GAD-7^1)^					**< 0.001**
< 5	1,266 (80.0)	1,534 (84.6)	1,432 (85.4)	4,232 (83.6)	
≥ 5	285 (20.0)	265 (15.4)	235 (14.6)	785 (16.5)	
HINT-8^2)^					**< 0.001**
High	1,289 (85.9)	1,655 (93.2)	1,575 (95.8)	4,519 (92.0)	
Low	262 (14.1)	144 (6.8)	92 (4.2)	498 (8.0)	
Chronic conditions					**< 0.001**
< 2	1,059 (73.2)	1,356 (79.0)	1,369 (85.2)	3,784 (79.5)	
≥ 2	492 (26.9)	443 (21.0)	298 (14.9)	1,233 (20.5)	

Data are expressed as count (percentage) using the χ2 test.

Bold values indicate p < 0.05.

GAD-7, generalized anxiety disorder-7; HINT-8, Health-related Quality of Life Instrument with 8 items, developed to assess HRQoL in the Korean population.

Higher HL was more prevalent among females; individuals with higher educational attainment; those in the highest income quartile; current smokers; alcohol consumers; married individuals; participants meeting aerobic physical activity guidelines; those without recent outpatient or inpatient health care utilization; and those with a lower chronic disease burden.

The prevalence of anxiety symptoms, defined as a GAD-7 score ≥ 5, was highest in the low HL group (20.0%), compared with the middle (15.4%) and high HL groups (14.6%) (p < 0.001). Similarly, the prevalence of low HRQoL was greater among participants with low HL (14.1%) than among those with high HL (4.2%) (p < 0.001).

### Association between HL, anxiety symptoms, and HRQoL

Table 2 shows that the low (OR: 1.93; 95% CI: 1.52–2.46; p < 0.001) and middle HL (OR: 1.30, 95% CI: 1.04–1.62; p = 0.024) groups had significantly higher odds of anxiety symptoms than the high HL group. Lower HL was associated with a reduced likelihood of good HRQoL; the low HL group showed significantly lower HRQoL than the high HL group (OR: 0.49; 95% CI: 0.36–0.66; p < 0.001). The middle HL group showed a nonsignificant trend toward lower HRQoL (OR: 0.77; 95% CI: 0.56–1.06; p = 0.115). In addition, sex, smoking status, healthcare utilization, and chronic disease burden were significantly associated with both anxiety symptoms and HRQoL.

**Table 2:** Multivariable logistic regression for generalized anxiety symptoms and health-related quality of life by health literacy level.

	GAD-7^1)^		HINT-8^2)^	
	OR (95% CI)	p-value	OR (95% CI)	p-value
HL (ref = High)				
Middle HL	1.30 (1.04–1.62)	**0.024**	0.77 (0.56–1.06)	0.115
Low HL	1.93 (1.52–2.46)	**< 0.001**	0.49 (0.36–0.66)	**< 0.001**
Age (ref = 19–39 years)				
40–64	0.62 (0.47–0.81)	**0.001**	0.83 (0.54–1.27)	0.386
≥ 65	0.40 (0.27–0.57)	**< 0.001**	0.65 (0.42–1.02)	0.060
Sex (ref = female)				
Male	0.50 (0.41–0.60)	**< 0.001**	2.35 (1.73–3.19)	**< 0.001**
Smoking				
Yes	1.85 (1.42–2.43)	**< 0.001**	0.58 (0.41–0.83)	**0.003**
Alcohol consumption				
Yes	1.02 (0.81–1.27)	0.872	1.27 (0.98–1.64)	0.072
Household income (ref = Low)				
Low-intermediate	0.76 (0.55–1.05)	0.097	1.69 (1.24–2.30)	**0.001**
Intermediate	0.64 (0.47–0.87)	**0.005**	2.34 (1.60–3.42)	**< 0.001**
Upper-intermediate	0.67 (0.47–0.95)	**0.023**	2.80 (1.83–4.29)	**< 0.001**
Marital status				
Married	0.77 (0.59–1.00)	0.054	1.36 (0.84–2.20)	0.209
Aerobic physical activity				
Yes	1.01 (0.84–1.22)	0.928	1.43 (1.13–1.81)	**0.003**
Education (ref = <high school)				
High school	1.23 (0.90–1.70)	0.206	1.65 (1.20–2.26)	**0.002**
≥ College	1.47 (1.04–2.07)	**0.027**	1.82 (1.29–2.58)	**0.001**
Inpatient visits				
Yes	1.42 (1.03–1.95)	**0.031**	0.40 (0.30–0.54)	**< 0.001**
Outpatient visits				
Yes	1.43 (1.15–1.78)	**0.001**	0.57 (0.45–0.71)	**< 0.001**
Chronic conditions				
≥ 2	1.30 (1.01–1.67)	**0.042**	0.73 (0.57–0.94)	**0.014**

OR, odds ratio; CI, confidence interval; ref, reference

Bold values indicate p < 0.05.

GAD-7, generalized anxiety disorder-7; HINT-8, Health-related Quality of Life Instrument with 8 Items, developed to assess HRQoL in the Korean population.

### Associations between HL and anxiety symptoms by age group

As shown in Table 3, low HL was associated with higher odds of anxiety symptoms among young adults (OR: 2.34; 95% CI: 1.62–3.37; p < 0.001) and middle-aged adults (OR: 1.92; 95% CI: 1.34–2.75; p < 0.001). In contrast, no significant association was observed among older adults (aged ≥ 65 years).

**Table 3:** Associations between health literacy levels and generalized anxiety symptoms by age group.

	Anxiety symptoms					
	Young adults (19–39 years)		Middle-aged adults (40–64 years)		Older adults (≥ 65 years)	
	OR (95% CI)	p-value	OR (95% CI)	p-value	OR (95% CI)	p-value
HL (ref = High)						
Middle HL	1.23 (0.88–1.73)	0.226	1.40 (0.98–2.00)	0.061	1.11 (0.63–1.94)	0.712
Low HL	2.34 (1.62–3.37)	**< 0.001**	1.92 (1.34–2.75)	**< 0.001**	1.19 (0.66–2.15)	0.550
Sex (ref = female)						
Male	0.44 (0.32–0.59)	**< 0.001**	0.56 (0.40–0.79)	**0.001**	0.58 (0.37–0.89)	**0.014**
Smoking						
Yes	1.70 (1.13–2.55)	**0.011**	1.93 (1.28–2.90)	**0.002**	2.42 (1.29–4.57)	**0.006**
Alcohol consumption						
Yes	0.99 (0.73–1.36)	0.967	1.09 (0.79–1.50)	0.591	0.88 (0.57–1.35)	0.547
Household income (ref = Low)						
Low-intermediate	1.02 (0.55–1.92)	0.939	0.53 (0.33–0.86)	**0.011**	0.76 (0.49–1.18)	0.217
Intermediate	0.86 (0.49–1.51)	0.588	0.42 (0.25–0.69)	**0.001**	0.73 (0.42–1.27)	0.260
Upper-intermediate	0.80 (0.43–1.49)	0.473	0.54 (0.34–0.87)	**0.012**	0.84 (0.39–1.82)	0.655
Marital status						
Married	0.76 (0.56–1.03)	0.074	0.72 (0.44–1.19)	0.202	2.94 (0.34–25.37)	0.324
Aerobic physical activity						
Yes	0.96 (0.73–1.26)	0.768	1.02 (0.75–1.40)	0.884	1.32 (0.87–1.99)	0.189
Education (ref =< High school)						
High school	1.75 (0.65–4.75)	0.267	1.27 (0.79–2.04)	0.330	0.91 (0.56–1.47)	0.686
≥ College	2.00 (0.73–5.47)	0.174	1.47 (0.88–2.48)	0.142	0.96 (0.51–1.81)	0.892
Inpatient visits						
Yes	1.64 (1.04–2.59)	**0.034**	1.16 (0.71–1.90)	0.559	1.39 (0.81–2.39)	0.225
Outpatient visits						
Yes	1.68 (1.20–2.35)	**0.003**	1.34 (0.97–1.86)	0.075	1.10 (0.73–1.67)	0.639
Chronic conditions						
≥ 2	1.61 (0.84-3.11)	0.152	1.27 (0.91–1.76)	0.160	1.24 (0.83–1.84)	0.292

OR, odds ratio; CI, confidence interval; ref, reference

Bold values indicate p < 0.05.

Anxiety symptoms were assessed using the GAD-7 scale. GAD-7, generalized anxiety disorder-7

### Associations between HL and HRQoL by age group

As shown in Table 4, both low (OR: 0.30; 95% CI: 0.15–0.58; p < 0.001) and middle HL (OR: 0.53; 95% CI: 0.28–0.99; p = 0.049) were significantly associated with poorer HRQoL among young adults (19–39 years).

**Table 4:** Associations between health literacy levels and health-related quality of life by age group.

	HRQoL					
	Young adults (19–39 years)		Middle-aged adults (40–64 years)		Older adults (≥ 65 years)	
	OR (95% CI)	p-value	OR (95% CI)	p-value	OR (95% CI)	p-value
HL (ref = High)						
Middle HL	0.53 (0.28–0.99)	**0.049**	0.79 (0.45–1.39)	0.415	1.18 (0.69–2.01)	0.544
Low HL	0.30 (0.15–0.58)	**< 0.001**	0.68 (0.37–1.25)	0.209	0.58 (0.34–0.97)	**0.040**
Sex (ref = female)						
Male	4.65 (2.29–9.44)	**< 0.001**	1.74 (1.01–2.98)	**0.045**	1.81 (1.29–2.55)	**0.001**
Smoking						
Yes	0.38 (0.20–0.74)	**0.005**	0.70 (0.41–1.20)	0.194	0.72 (0.41–1.27)	0.255
Alcohol consumption						
Yes	1.05 (0.65–1.71)	0.838	1.33 (0.82–2.17)	0.247	1.45 (0.94–2.23)	0.091
Household income (ref = Low)						
Low-intermediate	1.63 (0.67–3.96)	0.277	2.20 (1.26–3.84)	**0.006**	1.51 (1.01–2.24)	**0.044**
Intermediate	2.39 (0.96–5.99)	0.062	3.39 (1.81–6.34)	**< 0.001**	1.69 (1.01–2.82)	**0.045**
Upper-intermediate	2.89 (1.06–7.86)	**0.038**	3.92 (1.88–8.19)	**< 0.001**	2.20 (1.23–3.96)	**0.009**
Marital status						
Married	1.61 (0.90–2.89)	0.111	1.57 (0.69–3.59)	0.280	0.54 (0.10–2.91)	0.473
Aerobic physical activity						
Yes	1.79 (1.06–2.99)	**0.028**	1.12 (0.75–1.69)	0.576	1.43 (1.00–2.05)	0.050
Education (ref = <High school)						
High school	1.52 (0.30–7.81)	0.613	1.18 (0.70–1.99)	0.533	1.92 (1.24–2.95)	**0.003**
≥ College	0.86 (0.17–4.29)	0.850	2.41 (1.32–4.38)	**0.004**	2.74 (1.36–5.52)	**0.005**
Inpatient visits						
Yes	0.29 (0.17–0.51)	**< 0.001**	0.52 (0.33–0.81)	**0.004**	0.44 (0.28–0.68)	**< 0.001**
Outpatient visits						
Yes	0.74 (0.42–1.30)	0.295	0.45 (0.31–0.66)	**< 0.001**	0.61 (0.44–0.84)	**0.003**
Chronic conditions						
≥ 2	0.33 (0.11–1.04)	0.058	0.54 (0.35–0.84)	**0.007**	1.06 (0.80–1.42)	0.668

OR, odds ratio; CI, confidence interval; ref, reference

Bold values indicate p < 0.05.

HRQoL was assessed using the HINT-8 scale. HINT-8, Health-related Quality of Life Instrument with 8 Items, developed to assess HRQoL in the Korean population.

No significant association between HL and HRQoL was observed among middle-aged adults (40–64 years). In contrast, among older adults (≥ 65 years), low HL was significantly associated with poorer HRQoL (OR: 0.58; 95% CI: 0.34–0.97; p = 0.040).

## Discussion

In this study, we analyzed a nationally representative sample of Korean adults and found that lower HL was significantly associated with higher odds of anxiety symptoms and lower HRQoL. Age-stratified analyses further demonstrated that these associations differed across young, middle-aged, and older adults.

These findings are consistent with previous evidence linking lower HL to higher levels of anxiety [11].

For example, the EUROASPIRE V multinational cross-sectional survey of patients with coronary heart disease reported a significant association between lower HL and higher anxiety levels [43]. Similarly, a cross-sectional descriptive-analytical study conducted in Khuzestan, Iran, among adults aged 18–65 years found that lower HL was significantly associated with increased anxiety symptoms [16].

The HRQoL findings also align with those reported in previous studies. A cross-sectional study in Germany reported that low HL was associated with poorer HRQoL [44], while a study of Korean adults with chronic diseases found that adequate HL was associated with higher HRQoL [7]. However, studies involving patients with heart failure have not consistently identified a significant association between HL and HRQoL, indicating that this relationship remains inconclusive [24]. Therefore, the present study, conducted in a nationally representative adult population, contributes to a broader understanding of the inconsistent findings reported in previous studies. Additional evidence of age-specific differences in the associations among HL, anxiety symptoms, and HRQoL, was also provided.

Among young adults, lower HL was associated with higher odds of anxiety symptoms and poorer HRQoL. Young adulthood is characterized by social identity formation, career exploration, and the pursuit of financial independence, all of which may expose individuals to significant psychosocial stressors [45]. These challenges may be particularly difficult for individuals with low HL [46]. In Korea, suicide has been reported as the leading cause of death among individuals aged 20–30 years, highlighting the vulnerability of this age group with respect to mental health [47]. Moreover, in the present study, we used data from 2023, reflecting the post–COVID–19 socioeconomic context. The prolonged pandemic contributed to economic contraction and employment instability, intensifying job insecurity among young adults [48,49]. Employment rates have gradually recovered worldwide; however, Korea’s employment rate remains slightly below the Organization for Economic Co-operation and Development average. In addition, corporate downsizing and employer preferences for experienced workers have further limited labor market entry for younger individuals [49,50]. The post-pandemic surge in housing prices has also widened wealth disparities between homeowners and non-homeowners, disproportionately affecting individuals in their 20s and 30s and exacerbating economic insecurity [51]. Within this context, young adults with limited HL may lack the capacity to effectively respond to employment instability and economic shocks, which may partly explain their heightened risk of anxiety symptoms.

In middle-aged adults, HL was significantly associated with anxiety symptoms, whereas no significant relationship was observed between HL and HRQoL.

To date, few studies have examined age-specific associations between HL and anxiety symptoms. Middle adulthood is typically characterized by the accumulation of multiple life stressors, including workplace demands, family caregiving responsibilities, and emerging health problems, all of which have been significantly associated with declines in mental health and overall well-being [52,53]. In this context, these stressors may have exerted a stronger influence on HRQoL than HL among middle-aged adults in this study, which may partly explain why HL was associated with anxiety symptoms but not with HRQoL in this population.

Among older adults, no significant association was observed between HL and anxiety symptoms. This finding aligns with that of previous studies reporting no link between HL and anxiety symptoms in older populations [54]. The absence of a significant relationship may suggest that anxiety in older adults is more significantly influenced by factors such as declines in physical function, greater chronic disease burden, and social isolation than by HL [13]. Moreover, previous studies have demonstrated that older adults tend to underreport affective symptoms, including dysphoria and anhedonia, compared with younger adults [55], which may further attenuate the observed association between HL and anxiety symptoms in this age group. In contrast, a significant positive association was observed between HL and HRQoL among older adults. Similarly, a domestic study of older adults with mild cognitive impairment [17] reported a significant positive association between HL and HRQoL, suggesting that HL may be a key determinant of quality of life in this population.

Several mechanisms have been proposed in the literature to explain the associations among HL, anxiety symptoms, and HRQoL.

First, limited HL may directly contribute to increased anxiety symptoms and reduced HRQoL. Individuals with low HL may experience difficulty in understanding essential health information, which has been associated with greater psychological stress and anxiety [16]. Furthermore, HL is closely associated with effective self-management and the ability to cope with illness [56]. Consequently, limited HL may impair an individual’s capacity to manage their health, leading to poorer HRQoL [18].

Second, low HL may indirectly influence mental health and HRQoL by increasing the risk of chronic disease. Consistent with previous studies [57], we showed that individuals with low HL had a greater number of physician-diagnosed chronic conditions, particularly two or more conditions, and were more likely to experience anxiety symptoms and lower HRQoL. These findings suggest that low HL is associated with a higher chronic disease burden, which may partly explain its association with anxiety symptoms and HRQoL.

Third, HL has been associated with patterns of healthcare utilization and treatment adherence, which may contribute to delayed care and higher symptom burden.

In patients with hypertension, a community-based study reported that higher HL was significantly associated with better medication adherence [58], supporting the notion that improving HL may enhance adherence and timely care. In contrast, limited HL may hinder timely medical access and early intervention [11], potentially exacerbating the severity of anxiety symptoms. For example, despite the high prevalence of anxiety disorders, a recent population-based study in Singapore reported a median treatment delay of 9 years, with over half of individuals with anxiety disorders experiencing delayed treatment initiation [59].

Taken together, improving overall HL may help reduce delays in treatment and facilitate earlier interventions, which may, in turn, alleviate anxiety symptoms.

This study has several notable strengths. First, the use of nationally representative data from KNHANES addresses limitations of previous studies restricted to specific patient groups, older adults, or adolescents, thereby enhancing the generalizability of the findings.

Second, in this study, we provide a more comprehensive understanding of the relationship between HL, anxiety symptoms, and HRQoL by examining these outcomes simultaneously. Finally, age-stratified analyses identified differences in the associations of HL across the life course, providing essential evidence that HL may influence anxiety symptoms and HRQoL differently across age groups.

## Study limitations

This study has certain limitations. First, although the analytical sample of approximately 5,000 participants ensured national representativeness, a significant number of participants were excluded due to missing covariate data. Excluding individuals with incomplete information may have introduced selection bias if excluded participants differed systematically from those included in the final analytical sample. Because the characteristics of excluded and included participants were not formally compared, the possibility of selection bias arising from a complete-case analysis cannot be fully excluded. In addition, the final sample size may not have been sufficient for detailed subgroup analyses, particularly in age-stratified models, potentially limiting the precision of estimates and statistical power within each age group.

Second, the cross-sectional design precludes causal inference. Significant associations were observed; however, they should not be interpreted as causal relationships. Reverse causation is also possible; for example, poorer mental health or lower HRQoL may limit an individual’s ability to access, understand, or apply health information. Moreover, as depression and perceived stress were not assessed in the 2023 KNHANES, adjustment for these key mental health variables was not feasible. The absence of these measures raises the potential for residual confounding.

Future longitudinal or interventional studies are needed to clarify the directionality and causal pathways of these associations.

Third, data were collected via self-reported questionnaires, which may have introduced recall and reporting biases. However, KNHANES employs trained interviewers who conduct face-to-face interviews, which likely mitigates some limitations of self-report data and enhances the reliability of responses.

Finally, we relied on data from a single survey year, limiting the ability to examine temporal changes or long-term trends. Future studies using multi-year or longitudinal data are crucial to address this limitation.

## Conclusions

In this study, we examined the associations among HL, anxiety symptoms, and HRQoL in Korean adults. Lower HL was associated with significantly higher odds of anxiety symptoms and poorer HRQoL, with these associations most evident among young adults.

These findings suggest that HL may serve as an important social determinant of mental health and quality of life. Public health strategies to improve HL may help reduce anxiety symptoms and enhance HRQoL, particularly when interventions account for age-specific risk factors and apply tailored approaches.

## References

[1] Organization WH. Health literacy. 2024.

[2] Coughlin SS, Vernon M, Hatzigeorgiou C, George V. Health Literacy, Social Determinants of Health, and Disease Prevention and Control. J Environ Health Sci. 2020;6(1). PMC7889072 33604453

[3] Cho YI, Lee S-YD, Arozullah AM, Crittenden KS. Effects of health literacy on health status and health service utilization amongst the elderly. Soc Sci Med. 2008;66(8):1809–16. doi: 10.1016/j.socscimed.2008.01.003 18295949

[4] Song I, Lee SH. COVID-19 vaccine refusal associated with health literacy: findings from a population-based survey in Korea. BMC Public Health. 2023;23(1):255. doi: 10.1186/s12889-023-15182-0 36747179 PMC9900554

[5] Palumbo R, Annarumma C, Adinolfi P, Musella M, Piscopo G. The Italian Health Literacy Project: Insights from the assessment of health literacy skills in Italy. Health Policy. 2016;120(9):1087–94. doi: 10.1016/j.healthpol.2016.08.007 27593949

[6] Puente-Maestu L, Calle M, Rodríguez-Hermosa JL, Campuzano A, de Miguel Díez J, Álvarez-Sala JL, et al. Health literacy and health outcomes in chronic obstructive pulmonary disease. Respir Med. 2016;115:78–82. doi: 10.1016/j.rmed.2016.04.016 27215508

[7] Song I. Relationship between health literacy and health-related quality of life in Korean adults with chronic diseases. PLoS One. 2024;19(4):e0301894. doi: 10.1371/journal.pone.0301894 38635779 PMC11025905

[8] Yoon J, Kang SJ, Lee M, Cho J. Development and validation of the Health Literacy Index for the Community for the Korean National Health and Nutrition and Examination Survey. Epidemiol Health. 2024;46:e2024061. doi: 10.4178/epih.e2024061 39026432 PMC11826031

[9] Paasche-Orlow MK, Parker RM, Gazmararian JA, Nielsen-Bohlman LT, Rudd RR. The prevalence of limited health literacy. J Gen Intern Med. 2005;20(2):175–84. doi: 10.1111/j.1525-1497.2005.40245.x 15836552 PMC1490053

[10] Sørensen K, Pelikan JM, Röthlin F, Ganahl K, Slonska Z, Doyle G, et al. Health literacy in Europe: comparative results of the European health literacy survey (HLS-EU). Eur J Public Health. 2015;25(6):1053–8. doi: 10.1093/eurpub/ckv043 25843827 PMC4668324

[11] Magallón-Botaya R, Méndez-López F, Oliván-Blázquez B, Carlos Silva-Aycaguer L, Lerma-Irureta D, Bartolomé-Moreno C. Effectiveness of health literacy interventions on anxious and depressive symptomatology in primary health care: A systematic review and meta-analysis. Front Public Health. 2023;11:1007238. doi: 10.3389/fpubh.2023.1007238 36844856 PMC9948257

[12] Yang Y, Miao L, Liu X, Su W, Liu S. The impact of mental health literacy on depression, anxiety and well-being among vocational nursing students: mediating roles of resilience. Front Psychiatry. 2025;16:1585642. doi: 10.3389/fpsyt.2025.1585642 40661886 PMC12256488

[13] Zhong S-L, Wang S-B, Ding K-R, Tan W-Y, Zhou L. Low mental health literacy is associated with depression and anxiety among adults: a population-based survey of 16,715 adults in China. BMC Public Health. 2024;24(1):2721. doi: 10.1186/s12889-024-20020-y 39370527 PMC11456243

[14] Ding K-R, Wang S-B, Xu W-Q, Lin L-H, Liao D-D, Chen H-B, et al. Low mental health literacy and its association with depression, anxiety and poor sleep quality in Chinese elderly. Asia Pac Psychiatry. 2022;14(4):e12520. doi: 10.1111/appy.12520 36210054

[15] Haeri-Mehrizi A, Mohammadi S, Rafifar S, Sadighi J, Kermani RM, Rostami R, et al. Health literacy and mental health: a national cross-sectional inquiry. Sci Rep. 2024;14(1):13639. doi: 10.1038/s41598-024-64656-7 38871848 PMC11176292

[16] Dadgarinejad A, Nazarihermoshi N, Hematichegeni N, Jazaiery M, Yousefishad S, Mohammadian H, et al. Relationship between health literacy and generalized anxiety disorder during the COVID-19 pandemic in Khuzestan province, Iran. Frontiers in Psychology. 2024;14. 10.3389/fpsyg.2023.1294562 PMC10811604 38282836

[17] Kim CY, Choi B-Y, Ryoo S-W, Son S-Y, Min J-Y, Min K-B. Health Literacy and Health-Related Quality of Life in Older Adults with Mild Cognitive Impairment. J Am Med Dir Assoc. 2024;25(11):105253. doi: 10.1016/j.jamda.2024.105253 39265633

[18] Rague JT, Kim S, Hirsch J, Meyer T, Rosoklija I, Larson JE, et al. The Association of Health Literacy with Health-Related Quality of Life in Youth and Young Adults with Spina Bifida: A Cross-Sectional Study. J Pediatr. 2022;251:156-163.e2. doi: 10.1016/j.jpeds.2022.08.005 35970239 PMC9843738

[19] Study protocol for the World Health Organization project to develop a Quality of Life assessment instrument (WHOQOL). Qual Life Res. 1993;2(2):153–9. doi: 10.1007/bf00435734 8518769

[20] Wang C, Li H, Li L, Xu D, Kane RL, Meng Q. Health literacy and ethnic disparities in health-related quality of life among rural women: results from a Chinese poor minority area. Health Qual Life Outcomes. 2013;11:153. doi: 10.1186/1477-7525-11-153 24020618 PMC3847672

[21] Sertkaya Z, Koyuncu E, Nakipoğlu Yüzer GF, Özgirgin N. Investigation of health literacy level and its effect on quality of life in patients with spinal cord injury. J Spinal Cord Med. 2023;46(1):62–7. doi: 10.1080/10790268.2021.1991162 34726584 PMC9897774

[22] Xia J, Wu P, Deng Q, Yan R, Yang R, Lv B, et al. Relationship between health literacy and quality of life among cancer survivors in China: a cross-sectional study. BMJ Open. 2019;9(12):e028458. doi: 10.1136/bmjopen-2018-028458 31892642 PMC6955568

[23] Sayah FA, Qiu W, Johnson JA. Health literacy and health-related quality of life in adults with type 2 diabetes: a longitudinal study. Qual Life Res. 2016;25(6):1487–94. doi: 10.1007/s11136-015-1184-3 26603739

[24] Zhang J, Gilmour S, Liu Y, Ota E. Effect of health literacy on quality of life among patients with chronic heart failure in China. Qual Life Res. 2020;29(2):453–61. doi: 10.1007/s11136-019-02332-4 31628646

[25] Nguyen HC, Nguyen MH, Do BN, Tran CQ, Nguyen TTP, Pham KM, et al. People with Suspected COVID-19 Symptoms Were More Likely Depressed and Had Lower Health-Related Quality of Life: The Potential Benefit of Health Literacy. J Clin Med. 2020;9(4):965. doi: 10.3390/jcm9040965 32244415 PMC7231234

[26] Alinejad-Tilaki A, Omidvar S, Kheirkhah F, Bakhtiari A, Gholinia H. Health literacy and its relationship with mental health and quality of life in freshmen students. BMC Public Health. 2025;25(1):106. doi: 10.1186/s12889-024-21202-4 39789508 PMC11715539

[27] Lee MK, Oh J. Health-Related Quality of Life in Older Adults: Its Association with Health Literacy, Self-Efficacy, Social Support, and Health-Promoting Behavior. Healthcare (Basel). 2020;8(4):407. doi: 10.3390/healthcare8040407 33081352 PMC7712387

[28] Liu S, Meng Z, Wang S, Wang H, Fan D, Wu M, et al. The role of anxiety in the association between nutrition literacy and health-related quality of life among college students. Sci Rep. 2024;14(1):24618. doi: 10.1038/s41598-024-76361-6 39427070 PMC11490534

[29] Lorini C, Lastrucci V, Paolini D, Bonaccorsi G, Florence Health Literacy Research Group. Measuring health literacy combining performance-based and self-assessed measures: the roles of age, educational level and financial resources in predicting health literacy skills. A cross-sectional study conducted in Florence (Italy). BMJ Open. 2020;10(10):e035987. doi: 10.1136/bmjopen-2019-035987 33020080 PMC7537461

[30] Guggiari E, Jaks R, Berger FMP, Nicca D, De Gani SM. Health Literacy in the Canton of Zurich: First Results of a Representative Study. Int J Environ Res Public Health. 2021;18(23):12755. doi: 10.3390/ijerph182312755 34886479 PMC8657543

[31] Okan O, Bollweg TM, Berens EM, Hurrelmann K, Bauer U, Schaeffer D. Coronavirus-related health literacy: a cross-sectional study in adults during the COVID-19 infodemic in Germany. Int J Environ Res Public Health. 2020;17(15). 10.3390/ijerph17155503 PMC7432052 32751484

[32] Svendsen MT, Bak CK, Sørensen K, Pelikan J, Riddersholm SJ, Skals RK, et al. Associations of health literacy with socioeconomic position, health risk behavior, and health status: a large national population-based survey among Danish adults. BMC Public Health. 2020;20(1):565. doi: 10.1186/s12889-020-08498-8 32345275 PMC7187482

[33] Yoon J, Cho J, Kang S, Oh K, Choi S, Kang Y. Development of Health Literacy Index for The Korea National Health and Nutrition Examination Survey. Jugan Geongang Gwa Jilbyeong. 2023;16(23):709–25. doi: 10.56786/PHWR.2023.16.23.1 41334091 PMC12480063

[34] Dong M, Lu W, Zeng X, Yang Y, Liao D-D, Hou C-L, et al. Prevalence and correlates of generalized anxiety disorder and subthreshold anxiety symptoms in south China: A network perspective. J Affect Disord. 2025;379:232–40. doi: 10.1016/j.jad.2025.03.026 40068767

[35] Spitzer RL, Kroenke K, Williams JBW, Löwe B. A brief measure for assessing generalized anxiety disorder: the GAD-7. Arch Intern Med. 2006;166(10):1092–7. doi: 10.1001/archinte.166.10.1092 16717171

[36] Valuation JM. Valuation of Korean health-related quality of life instrument with 8 items (HINT-8). Cheongju: Korea Centers for Disease Control and Prevention. 2017.

[37] Kim W, Han K-T, Kim S. Health-related quality of life among cancer patients and survivors and its relationship with current employment status. Support Care Cancer. 2022;30(5):4547–55. doi: 10.1007/s00520-022-06872-3 35119519

[38] Jung SM, Lee M-R. Analyzing the effect of sleep duration, chronotype, and social jet lag on anxiety disorders and health-related quality of life: A cross-sectional study. PLoS One. 2024;19(11):e0314187. doi: 10.1371/journal.pone.0314187 39570939 PMC11581315

[39] Kang Y, Kang M, Lim H. Age-specific association between meal-skipping patterns and the risk of hyperglycemia in Korean adults: a national cross-sectional study using the KNHANES data. BMC Public Health. 2024;24(1):1697. doi: 10.1186/s12889-024-18762-w 38918764 PMC11201090

[40] Chen Q, Moore J, Noel L, von Sternberg K, Jones B. Sociodemographic Correlates of Low Health Literacy Skills Among Cancer Survivors: National Findings From BRFSS 2016. Am J Health Promot. 2024;38(6):757–66. doi: 10.1177/08901171231222073 38108189

[41] Kim CR, Jeon Y-J, Jeong T. Risk factors associated with low handgrip strength in the older Korean population. PLoS One. 2019;14(3):e0214612. doi: 10.1371/journal.pone.0214612 30921399 PMC6438516

[42] Wieczorek M, Meier C, Vilpert S, Reinecke R, Borrat-Besson C, Maurer J, et al. Association between multiple chronic conditions and insufficient health literacy: cross-sectional evidence from a population-based sample of older adults living in Switzerland. BMC Public Health. 2023;23(1):253. doi: 10.1186/s12889-023-15136-6 36747134 PMC9901105

[43] Jennings CS, Astin F, Prescott E, Hansen T, Gale Chris P, De Bacquer D. Illness perceptions and health literacy are strongly associated with health-related quality of life, anxiety, and depression in patients with coronary heart disease: results from the EUROASPIRE V cross-sectional survey. Eur J Cardiovasc Nurs. 2023;22(7):719–29. doi: 10.1093/eurjcn/zvac105 36351004

[44] Ehmann AT, Groene O, Rieger MA, Siegel A. The Relationship between Health Literacy, Quality of Life, and Subjective Health: Results of a Cross-Sectional Study in a Rural Region in Germany. Int J Environ Res Public Health. 2020;17(5):1683. doi: 10.3390/ijerph17051683 32150820 PMC7084276

[45] Babajide A, Ortin A, Wei C, Mufson L, Duarte CS. Transition Cliffs for Young Adults with Anxiety and Depression: Is Integrated Mental Health Care a Solution?. J Behav Health Serv Res. 2020;47(2):275–92. doi: 10.1007/s11414-019-09670-8 31428923 PMC7028507

[46] Lu Y, Li J, Cui Z, Zheng M, Zhao Y. Perceived stress and sleep quality in young and middle-aged patients with coronary heart disease: the mediating role of perceived social support and mental health literacy. Front Psychol. 2025;16:1444831. doi: 10.3389/fpsyg.2025.1444831 40510930 PMC12159011

[47] Jang H, Lee W, Kim Y-O, Kim H. Suicide rate and social environment characteristics in South Korea: the roles of socioeconomic, demographic, urbanicity, general health behaviors, and other environmental factors on suicide rate. BMC Public Health. 2022;22(1):410. doi: 10.1186/s12889-022-12843-4 35227243 PMC8887086

[48] Organization IL. World employment and social outlook: Trends 2023. Geneva: United Nations. 2023.

[49] Kwak Y, Kim Y, Chae H. Job search anxiety and flourishing among university students: the serial mediating effects of social support and strengths use. BMC Psychol. 2025;13(1):652. doi: 10.1186/s40359-025-02995-4 40597452 PMC12211445

[50] Ham S. Covid19 pandemic and the divided youth labor market in Korea. Korean Journal of Labor Studies. 2022;28(1):69–101.

[51] Ha T. COVID-19 and Household Wealth Heterogeneity: Evidence from South Korea. HPE-RPE. 2024;250(3):69–88. doi: 10.7866/hpe-rpe.24.3.3

[52] Gondek D, Bernardi L, McElroy E, Comolli CL. Why do Middle-Aged Adults Report Worse Mental Health and Wellbeing than Younger Adults? An Exploratory Network Analysis of the Swiss Household Panel Data. Appl Res Qual Life. 2024;19(4):1459–500. doi: 10.1007/s11482-024-10274-4 39211006 PMC11349807

[53] Gondek D, Lacey RE, Blanchflower DG, Patalay P. How is the distribution of psychological distress changing over time? Who is driving these changes? Analysis of the 1958 and 1970 British birth cohorts. Soc Psychiatry Psychiatr Epidemiol. 2022;57(5):1007–16. doi: 10.1007/s00127-021-02206-6 34807287 PMC9042977

[54] Sanftenberg L, Gschwendner M, Grass A, Rottenkolber M, Zöllinger I, Sebastiao M, et al. Associations of Mental Health Issues with Health Literacy and Vaccination Readiness against COVID-19 in Long-Term Care Facilities-A Cross-Sectional Analysis. Eur J Investig Health Psychol Educ. 2024;14(3):432–46. doi: 10.3390/ejihpe14030029 38534890 PMC10969694

[55] Lutz J, Van Orden KA. Sadness and Worry in Older Adults: Differentiating Psychiatric Illness from Normative Distress. Med Clin North Am. 2020;104(5):843–54. doi: 10.1016/j.mcna.2020.05.001 32773049 PMC7417641

[56] Lastrucci V, Lorini C, Caini S, Florence Health Literacy Research Group, Bonaccorsi G. Health literacy as a mediator of the relationship between socioeconomic status and health: A cross-sectional study in a population-based sample in Florence. PLoS One. 2019;14(12):e0227007. doi: 10.1371/journal.pone.0227007 31869381 PMC6927637

[57] Kwon DH, Kwon YD. Patterns of health literacy and influencing factors differ by age: a cross-sectional study. BMC Public Health. 2025;25(1):1556. doi: 10.1186/s12889-025-22838-6 40287654 PMC12032677

[58] Guo A, Jin H, Mao J, Zhu W, Zhou Y, Ge X, et al. Impact of health literacy and social support on medication adherence in patients with hypertension: a cross-sectional community-based study. BMC Cardiovasc Disord. 2023;23(1):93. doi: 10.1186/s12872-023-03117-x 36803662 PMC9940429

[59] Tan RHS, Koh YS, Vaingankar JA, Abdin E, Sambasivam R, Chong SA, et al. Treatment delays for mental disorders in Singapore: results from the Singapore Mental Health Study 2016. Soc Psychiatry Psychiatr Epidemiol. 2024;59(2):375–83. doi: 10.1007/s00127-023-02440-0 36786835

